# Compositional Analysis of Glycosaminoglycans in Different Lung Cancer Types—A Pilot Study

**DOI:** 10.3390/ijms24087050

**Published:** 2023-04-11

**Authors:** Domonkos Pál, Gábor Tóth, Simon Sugár, Kata Dorina Fügedi, Dániel Szabó, Ilona Kovalszky, Dávid Papp, Gitta Schlosser, Csaba Tóth, Tamás Tornóczky, László Drahos, Lilla Turiák

**Affiliations:** 1 MS Proteomics Research Group, Research Centre for Natural Sciences, H-1117 Budapest, Hungary; 2 Doctoral School of Pharmaceutical Sciences, Semmelweis University, H-1085 Budapest, Hungary; 3 Faculty of Chemical Technology and Biotechnology, Budapest University of Technology and Economics, H-1111 Budapest, Hungary; 4 1st Department of Pathology and Experimental Cancer Research, Semmelweis University, H-1085 Budapest, Hungary; 5 MTA-ELTE Lendület Ion Mobility Mass Spectrometry Research Group, ELTE Eötvös Loránd University, H-1117 Budapest, Hungary; 6 Hevesy György PhD School of Chemistry, ELTE Eötvös Loránd University, H-1117 Budapest, Hungary; 7 Teaching Hospital Markusovszky, University of Pécs, H-9700 Szombathely, Hungary; 8 Department of Pathology, Faculty of Medicine and Clinical Center, University of Pécs, H-7624 Pécs, Hungary

**Keywords:** glycosaminoglycan, chondroitin sulfate, heparan sulfate, adenocarcinoma, small cell carcinoma, squamous cell carcinoma, large cell carcinoma, lung cancer, mass spectrometry, HPLC

## Abstract

Lung cancer is one of the most commonly diagnosed cancer types. Studying the molecular changes that occur in lung cancer is important to understand tumor formation and identify new therapeutic targets and early markers of the disease to decrease mortality. Glycosaminoglycan chains play important roles in various signaling events in the tumor microenvironment. Therefore, we have determined the quantity and sulfation characteristics of chondroitin sulfate and heparan sulfate in formalin-fixed paraffin-embedded human lung tissue samples belonging to different lung cancer types as well as tumor adjacent normal areas. Glycosaminoglycan disaccharide analysis was performed using HPLC-MS following on-surface lyase digestion. Significant changes were identified predominantly in the case of chondroitin sulfate; for example, the total amount was higher in tumor tissue compared to the adjacent normal tissue. We also observed differences in the degree of sulfation and relative proportions of individual chondroitin sulfate disaccharides between lung cancer types and adjacent normal tissue. Furthermore, the differences in the 6-*O*-/4-*O*-sulfation ratio of chondroitin sulfate were different between the lung cancer types. Our pilot study revealed that further investigation of the role of chondroitin sulfate chains and enzymes involved in their biosynthesis is an important aspect of lung cancer research.

## 1. Introduction

Lung cancer (LC) is the second most frequently diagnosed cancer type and has the highest mortality of any type of tumor, because most cases are diagnosed at an advanced stage when the number of treatment options are limited and metastasis to distant organs has developed [1,2]. Based on histology, lung cancer can be classified into two main groups: small cell lung carcinoma (SCLC) and non-small cell lung carcinoma (NSCLC). NSCLC is responsible for approximately 85%, while SCLC for roughly 15% of LC cases [3]. NSCLC is a heterogeneous group of lung cancers and can be further categorized into the following main subtypes: adenocarcinoma (AC), squamous cell carcinoma (SqCC), and large cell carcinoma (LCC) [3,4,5]. There are many approaches to visualizing LC, such as imaging techniques like X-ray, CT scan, and PET scan, but the final diagnosis is confirmed by either the histological or cytological analysis of the biopsy sample. The therapy for SCLC and NSCLC strongly depends on the type of tumor and its progression and can include surgery, radio-chemotherapy, targeted treatment with antiangiogenic monoclonal antibodies or tyrosine kinase inhibitors, and immunotherapy [4,6].

Carbohydrate tumor markers are used as diagnostic biomarkers in various cancer types [7]. Glycosylation is one of the most frequent post-translational modifications, occuring when glycan units are added to proteins or other molecules, in glycoproteins most often through an oxygen or a nitrogen atom (*O*- and *N*-glycans) [8,9]. Glycosylation has a great impact on the functions of proteins, e.g., affecting various signaling events in the tumor microenvironment [10]. Changes in the glycosylation of proteins are a common phenomenon in cancer [11]. The increased proportion of branched *N*-glycans and the increased amount of *O*-glycans have already been reported in relation to lung cancer [8,12,13]. Proteoglycans (PGs), a specific class of glycoproteins, are macromolecules whose structure consists of a core protein and one or more glycosaminoglycan (GAG) chains covalently attached to it via a tetrasaccharide linker. PGs are predominantly located in the extracellular matrix and the plasma membrane. They play important roles in the stabilization of the extracellular matrix and signaling processes [9]. GAGs are linear polysaccharides, built up by repeating amino sugar and uronic acid or galactose building blocks. GAGs interact with growth factors and cytokines, thereby influencing the functions of cells [14]. GAG chains have a significant impact on the function of proteins due to their size and structural diversity, such as the sulfation patterns of the chains [15,16]. Sulfation along the chain is carried out by sulfotransferase enzymes [17]. Changes in the total quantity of GAG chains and in their sulfation patterns have been previously identified between healthy and tumor tissue [18,19,20]. Chondroitin sulfates (CS) and heparan sulfates (HS) are among the most important and most widely studied GAGs. The building blocks of CS chains are *N*-acetylgalactosamine (GalNAc) and glucoronic acid (GlcA), while HS chains consist of *N*-acetylglucosamine (GlcNAc) and glucuronic/iduronic acid (IdoA). The structure of CS and HS can be diverse since several possible sulfation positions exist, and the occupancy of these sites affects their functions. Both CS and HS chains have been previously analyzed with respect to lung cancer [21,22,23], however, the first studies only focused on comparing the total GAG content and different GAG classes between LC samples and normal tissues [21] and also between distinct LC types [22] in small sample cohorts (*n* = 11 and 13, respectively). HPLC-MS characterization of disaccharides originating from SqCC tissues and patient-matched normal tissue (*n* = 10) has only recently been performed [23], but comparison of different LC types at the disaccharide level has not been studied so far. In a recent comparative proteomics pilot study of SCLC, AC, SqCC, and LCC tissue samples, differences in the protein expression levels between the different tumor types and between tumor and tumor adjacent normal samples were identified [24]. Our aim was to study whether similar alterations occur at the GAG level as well. Therefore, the CS and HS compositions of different lung tumor phenotypes and tumor-adjacent normal lung tissue samples were investigated. The experiment was performed through disaccharide analysis following tissue surface digestion [25,26,27,28]. The disaccharides extracted from the tissue surface were desalted and then separated using capillary columns packed in-house with hydrophilic interaction liquid chromatography and weak anion exchange (HILIC-WAX) mixed mode resin and detected using negative ionization mass spectrometry. CS and HS disaccharides were analyzed using separate HPLC-MS methods developed recently; CS uses a 15 min gradient, while HS uses a slightly modified 25 min ammonium formate salt gradient [29,30,31].

## 2. Results

In the present study, we investigated CS and HS chains from formalin-fixed paraffin-embedded (FFPE) lung tissue. The samples originated from different lung cancer types (AC, SqCC, LCC, and SCLC) and adjacent normal tissue regions. Altogether, 77 CS (41 tumor, 36 tumor adjacent normal) and 76 HS (40 tumor, 36 tumor adjacent normal) samples were analyzed. The sample and patient information are summarized in Table 1. The presented results are based on the aforementioned sample numbers, but in some cases, there are minor deviations due to missing values. In the case of the total quantity and the total *N*/*O* ratio calculations, the missing values were replaced with zeros. In the event of significant changes, the exact sample numbers, the types of tests used, the fold change, and *p* values are listed in Supplementary Table S1 (CS cohort) and Supplementary Table S2 (HS cohort). The changes in the features of CS and HS GAGs were examined by analyzing the samples at the disaccharide level; the disaccharide structures can be found in Supplementary Tables S3 and S4.

**Figure 1:**
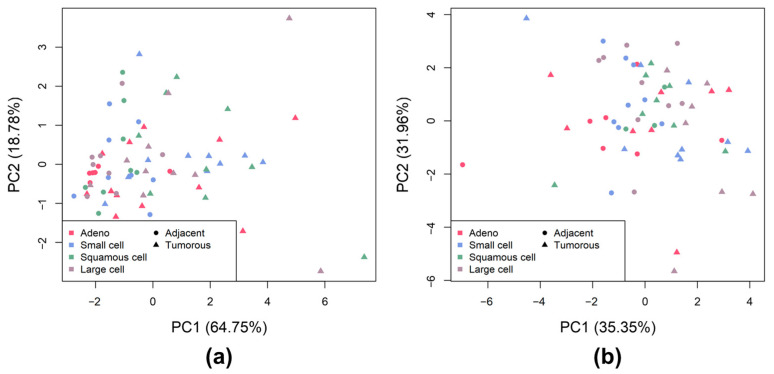
PCA of the analyzed samples (**a**): CS disaccharide abundances; (**b**): HS disaccharide abundances. Triangles and circles indicate tumor and tumor-adjacent samples, respectively. The different colors mark the different sample groups (pink for AC, blue for SCLC, green for SqCC, and grey for LCC).

First, principal component analysis (PCA) was performed separately for the CS and HS disaccharides detected (Figure 1) to visualize whether the tumor and tumor-adjacent samples and the lung tumor phenotypes can be distinguished from each other based on the disaccharide abundances. In the case of CS (Figure 1a), the tumor and tumor adjacent samples are slightly better separated than in the case of HS (Figure 1b), where they cannot be distinguished based on the abundance of HS disaccharides with high confidence. Furthermore, based on the PCA, the lung cancer phenotypes do not show large differences from each other based on the abundance of CS and HS disaccharides.

### 2.1. CS and HS Content and Sulfation Characteristics between All Tumor and All Tumor Adjacent Normal Samples

**Figure 2:**
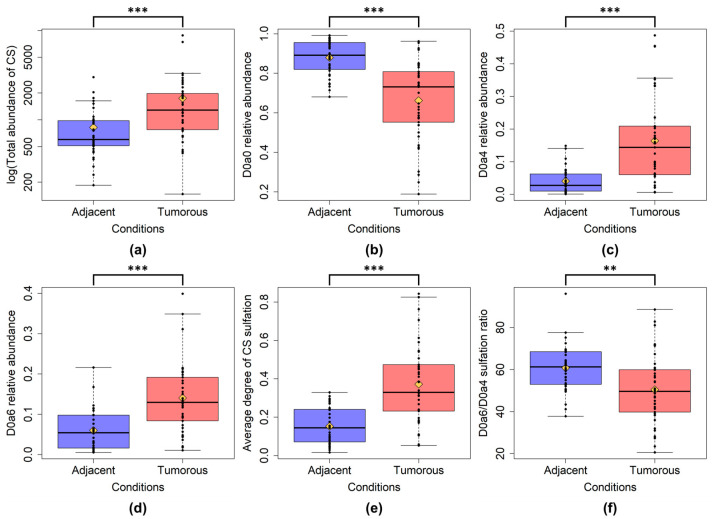
Box plots of altered CS characteristics between all adjacent and all tumor samples. (**a**): total CS quantity; (**b**): D0a0 relative abundance; (**c**): D0a4 relative abundance; (**d**): D0a6 relative abundance. (**e**): average degree of sulfation; (**f**): 6-*O*-/4-*O*-sulfation ratio. The yellow diamonds are the average values of the given sample groups. (**: *p* < 0.01; ***: *p* < 0.001).

The total CS disaccharide content was significantly higher in the tumor tissue samples compared to adjacent samples (Figure 2a). Examining the individual CS disaccharides between all the tumor and adjacent sample groups, the D0a0, D0a4, and D0a6 disaccharide contents were significantly different (Figure 2b–d). The D0a0 abundance decreased, while the D0a4 and D0a6 quantities increased in the tumor samples. The D0a10 doubly sulfated disaccharide had low abundance and did not show significant differences between any of the compared sample groups. Thus, its individual abundance will not be discussed further.

To identify the structural changes in GAGs, we examined the changes in sulfation patterns. Analyzing the CS sulfation between all tumor and all adjacent tissue samples, the average degree of sulfation and the 6-*O*/4-*O*-sulfation ratio changed significantly (Figure 2e,f). The average degree of sulfation was higher, while the 6-*O*-/4-*O*-sulfation ratio was lower in tumor samples.

**Figure 3:**
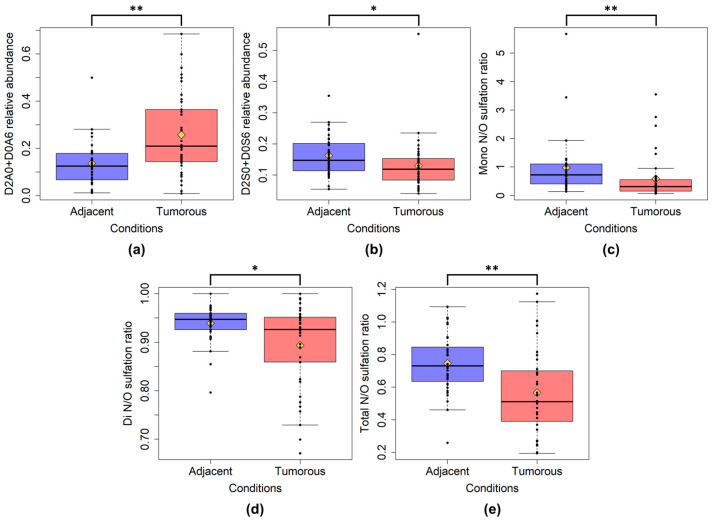
Box plots of altered HS characteristics between all adjacent and all tumor samples. (**a**): D2A0 + D0A6 relative abundance; (**b**): D2S0 + D0S6 relative abundance. (**c**): mono *N*/*O*-sulfation ratio; (**d**): di *N*/*O*-sulfation ratio; (**e**): total *N*/*O*-sulfation ratio. Mono-, di-, and total *N*/*O*-sulfation ratios were calculated as the ratio of the corresponding disaccharides weighted by their contribution to *O*- and *N*-sulfation (the number of the respective sulfate groups on each disaccharide). The yellow diamonds are the average values of the given sample groups. (*: *p* < 0.05; **: *p* < 0.01).

The total HS disaccharide content of the tumor samples showed a very similar value compared to the tumor-adjacent regions (Figure S1). To test this similarity, we used the Kolgomorov-Smirnov test (*p* = 0.9959). Comparing all the tumor and all the tumor-adjacent samples, the D2A0 + D0A6 and D2S0 + D0S6 HS disaccharides showed significant changes in their abundance (Figure 3a,b). The relative abundance of D2A0 + D0A6 was higher, while that of D2S0 + D0S6 was lower in the tumor regions. Analyzing the differences in HS sulfation between the tumor and adjacent samples, we observed a decrease in the mono-, di-, and total *N*/*O*-sulfation ratios in the case of the tumor samples (Figure 3c–e).

**Figure 4:**
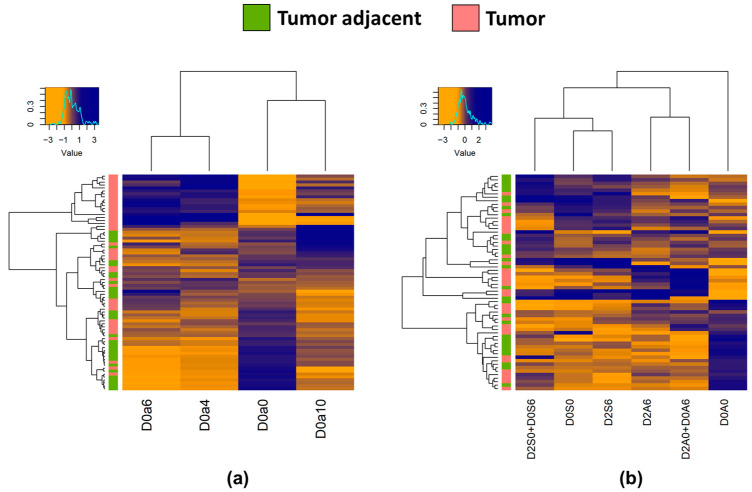
Heatmaps with hierarchical clustering of the (**a**): CS and (**b**): HS disaccharides between all adjacent and all tumor samples. The top left corner histogram shows correspondence between color hues and values.

Hierarchical clustering of tumor and tumor-adjacent samples showed that in the case of chondroitin sulfate, several tumor samples clustered together, while in the case of heparan sulfate, no clustering was observed (Figure 4).

### 2.2. CS and HS Content and Sulfation Characteristics between Tumor and Corresponding Tumor Adjacent Normal Samples

**Figure 5:**
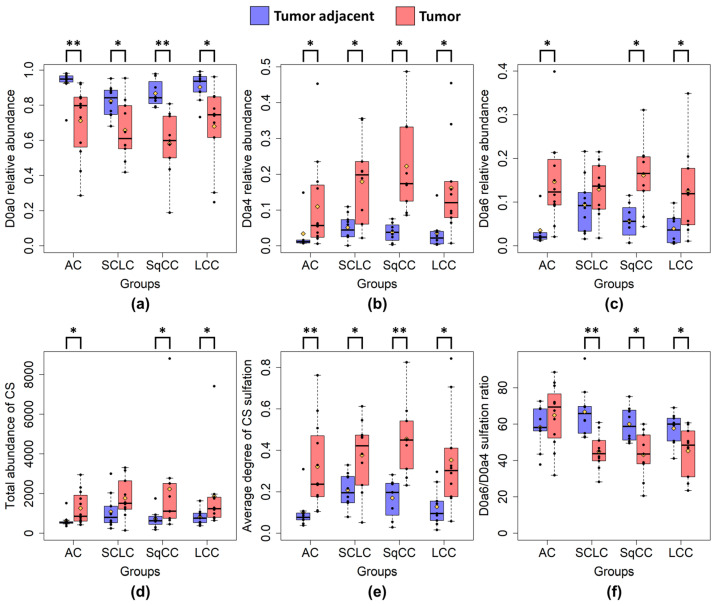
Altered CS sulfation characteristics between the tumor and adjacent samples. (**a**): D0a0 relative abundance; (**b**): D0a4 relative abundance; (**c**): D0a6 relative abundance; (**d**): total CS quantity; (**e**): average degree of CS sulfation; (**f**): 6-*O*-/4-*O*-sulfation ratio. Blue boxes represent tumor-adjacent normal samples, while red boxes represent tumor samples. (AC: adenocarcinoma; SCLC: small cell lung cancer; SqCC: squamous cell carcinoma; LCC: large cell carcinoma). The yellow diamonds are the average values of the given sample groups. (*: *p* < 0.05; **: *p* < 0.01).

Performing pairwise comparison between tumor samples and their respective adjacent normal groups, we found significant changes in CS sulfation level in the case of D0a0, D0a4, and D0a6 disaccharides, total quantity of the CS disaccharides, average degree of sulfation, and the 6-*O*/4-*O*-sulfation ratio (Figure 5a–f). The D0a0 content decreased, while the relative abundance of D0a4 and D0a6, and the average degree of sulfation, increased in all tumor types. The total CS quantity increased in the cases of AC, SqCC, LCC. The 6-*O*/4-*O*-sulfation ratio decreased in SCLC, SqCC, and LCC.

Performing pairwise comparisons between the tumor and the respective adjacent normal groups, the changes in HS sulfation levels and HS sulfation characteristics were not significant (Figures S2 and S3).

### 2.3. CS and HS Sulfation between Lung Tumor Phenotypes

**Figure 6:**
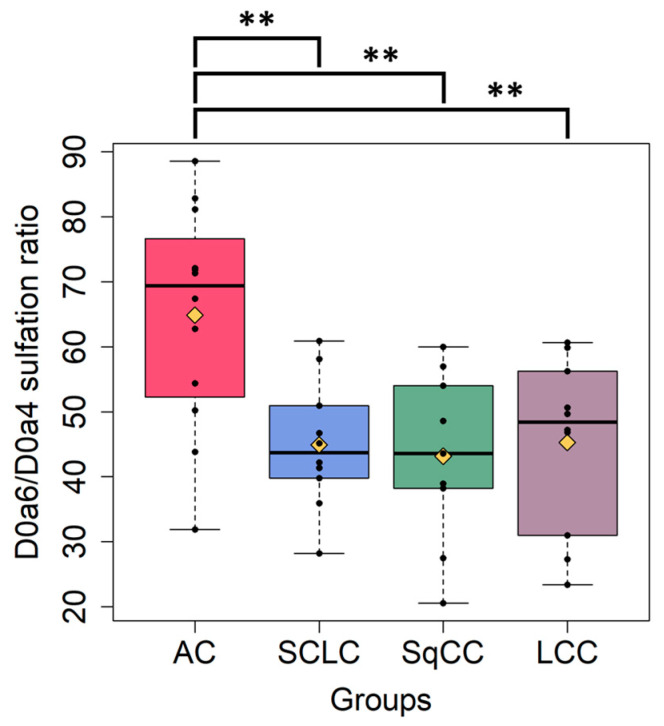
6-*O*-/4-*O*-sulfation ratio of the different lung cancer types analyzed (AC: adenocarcinoma, SCLC: small cell lung cancer, SqCC: squamous cell carcinoma, LCC: large cell carcinoma). The yellow diamonds are the average values of the given sample groups. (**: *p* < 0.01).

Comparing the different tumor types, the CS 6-*O*/4-*O*-sulfation ratio changed significantly between tumor groups. The 6-*O*/4-*O*-sulfation ratio of AC was significantly increased compared to the other tumor groups (Figure 6). Further CS sulfation characteristics between tumor types can be found in Supplementary Figure S4. In the case of HS, no significant differences were identified between the tumor types with regards to sulfation characteristics (Figures S5 and S6).

## 3. Discussion

In the present study, we analyzed the CS and HS disaccharide abundances and sulfation patterns of lung tissue samples from different types of lung tumors and tumor-adjacent normal samples. To the best of our knowledge, this is the first attempt to directly compare the sulfation characteristics of CS and HS GAGs in FFPE tissues of SCLC and different NSCLC (AC, SqCC, and LCC) subtypes.

The most critical part of our work was the tissue surface digestion of the FFPE tissue samples. The digestion, disaccharide extraction, and sample cleanup procedures have been thoroughly optimized, along with the HPLC-MS measurement methodology. The fully optimized protocols have recently been published [32].

Comparing all the tumor and all the adjacent samples, we could identify differences in the total CS disaccharide content and in the relative abundance of non-sulfated (D0a0) and mono-sulfated (D0a4 and D0a6) disaccharides. The mono-*O*-sulfated (D2A0 + D0A6) and di-sulfated (1-*O*- and 1-*N*-sulfated) (D2S0 + D0S6) HS disaccharides showed differences between the tumor and tumor adjacent samples. Analyzing the sulfation of CS samples, the average degree of sulfation and the 6-*O*-/4-*O*-sulfation ratio changed significantly between the tumor and adjacent normal samples. The mono-, di-, and total-*N/O*-sulfation levels of HS showed alterations between the tumor and tumor adjacent samples. Comparing the sulfation characteristics of CS between the tumor groups and their corresponding adjacent normal samples, the non-sulfated (D0a0), the mono-sulfated disaccharide (D0a4) levels, and the degree of sulfation changed in all tumor sample groups. 6-*O*-/4-*O*-sulfation ratio of CS disaccharides distinguished AC from all the other lung tumor types investigated.

A comparison of the total GAG content and individual GAG classes of lung carcinoma samples and tumor-distant normal tissues has already been performed several decades ago on a small number of samples (3 AC, 3 SqCC, and 5 control) [21]. Another study compared quantitative GAG changes in different LC types (2 SqCC, 4 AC, and 5 SCLC) [22]. These early studies used classical methods such as cellulose acetate electrophoresis as well as chemical and enzymatic digestion to determine the GAG abundance of the tissues. In line with these early results, we also found an increase in the total CS content for all the investigated LC types compared to the adjacent samples. A detailed study regarding the composition of GAGs and the subtype and degree of differentiation of lung carcinomas has been performed by analyzing 34 samples using histochemistry and spectrophotometry [33]. Total GAG content was higher in lung cancer phenotypes compared to normal lung tissue, and significant differences were also identified in the GAG fractions analyzed when comparing poorly differentiated and Clara cell type adenocarcinomas [33].

The disaccharide-specific analysis of lung cancer types has been mostly neglected to date. The most detailed results were reported by Li et al. on a small cohort of squamous cell carcinoma samples [23]. The authors used liquid chromatography and Western blotting to compare the structure of GAGs, glycolipids, and selected proteins in a cohort consisting of 10 SqCC and patient matched normal tissue. Although only SqCC tissues were analyzed, most of their observations are consistent with our results. They observed that the total HS content was not different between SqCC and normal tissue, while the CS content was two times higher in the case of SqCC. We also could not identify any significant difference in HS content between the investigated tumor and adjacent tissue samples, only in their CS content. Concerning HS disaccharides, Li et al. described that the trisulfated (D2S6), mono-*N*-sulfated (D0S0), and non-sulfated (D0A0) disaccharides were not different between normal and SqCC tissues, while the amount of di- (D0S6, D2S0), and mono-*O*- sulfated (D2A0) disaccharides decreased in tumor samples. In line with these observations, our results also show that the amount of tri- (D2S6), mono-*N*-sulfated (D0S0), and non-sulfated (D0A0) disaccharides does not change in the tumor tissue compared to adjacent regions. Furthermore, we also observed a decrease in the di-sulfated (D2S0, D0S6) disaccharides, but in contrast, the mono-sulfated (D2A0, D0A6) disaccharides increased significantly in the tumor samples. For CS, changes in the amount of mono-sulfated disaccharides were identified by Li et al. The level of D0a4 decreased, while D0a6 increased in SqCC tissues, while in our study both D0a4 and D0a6 increased in the different tumor types compared to the respective tumor adjacent tissue. Moreover, we observed a decrease of the non-sulfated (D0a0) component in all examined tumor tissue types relative to their adjacent tissue. These differential observations lay the basis for a future, larger-scale study and validation of results with complementary techniques.

Based on our previous results, several differences could be identified in the protein profiles of the different lung cancer types (SCLC, AC, SqCC, and LCC) using shotgun proteomics [24]. Proteoglycan core proteins identified included versican, which was overexpressed in AC, while perlecan, decorin, prolargin, and mimecan were underexpressed in SCLC and LCC; biglycan was underexpressed in AC, SCLC, and SqCC [24,34]. Versican and biglycan are CS proteoglycans. The increased expression of versican in AC is in correlation with our observation of the total CS GAG content. In a previous study, proteoglycan serglycin (SRGN) was shown to promote NSCLC cell migration [35]. NSCLC cells express SRGN, a heavily glycosylated protein that mainly contains CS- and fewer HS-GAG chains. The CS part of SRGN promotes binding to CD44 on the surface of tumor cells, which then supports cell migration. In the absence of CS chains, this does not occur [35].

Chondroitin sulfates play important biological roles in formation of the tumor niche. CS has been shown to be involved in cell-cell and cell-ECM interactions in solid tumors, promoting tumor cell adhesion as well as migration, leading to aggressive spread and metastasis [36]. Our results also revealed several significant changes in CS in relation to lung cancer. An increase in CS content has been observed in various tumor types, e.g., liver [20], prostate [19] highlighting the importance of CS in tumor formation. In our experiments, CS expression increased in tumor samples, implying the increased activity of enzymes involved in their formation.

Oncofetal CS, a placental CS type, has been shown to be expressed in cancer cells and tissues. The presence of oncofetal CS can be detected by the specific binding of the malarial VAR2CSA protein [37]. The minimal length of the CS-binding domain of the malarial VAR2CSA protein was identified in tumor and placental tissue as dodecasaccharide (dp12). This domain contains mostly 4-*O*-sulfated and some 6-*O*-sulfated *N*-acetylgalactosamine residues [37]. Accordingly, we could detect the increased abundance of D0a4 (AC, SCLC, SqCC, and LCC) and D0a6 (AC, SqCC, and LCC) sulfated CS components in the tumor tissue samples. Moreover, the 6-*O*-/4-*O*-sulfation ratio decreased between the tumor samples, except in AC. Based on these results, we can hypothesize the presence of oncofetal CS in the analyzed sample cohort as well. Furthermore, oncofetal CS expression was recently studied in lung cancer, and elevated levels were found to predict poor disease-free and overall survival in early-stage NSCLC independent of the presence of KRAS and EGFR mutations [38]. The results discussed above imply the necessity of future structural studies of CS disaccharides in different lung tumor types.

## 4. Materials and Methods

### 4.1. Chemicals

HPLC-MS-grade solvents were purchased from VWR Hungary (Debrecen, Hungary). Crystalline ammonium formate, ammonium bicarbonate, chondroitinase ABC, and formic acid (FA) were purchased from Merck (Budapest, Hungary). The Δ4,5-unsaturated chondroitin sulfate and heparan sulfate disaccharide standards and heparin lyase I-II-III enzymes were purchased from Iduron (Macclesfield, UK). Pierce graphite was purchased from Thermo Scientific. Cotton wool was purchased from DM—Drogerie Markt (Karlsruhe, Germany).

### 4.2. Tissue Sample Selection and Preparation

In this work, we analyzed adeno-, small cell lung-, squamous cell-, large cell lung- carcinoma, and tumor adjacent formalin-fixed, paraffin-embedded (FFPE) human tissue samples from the Department of Pathology, Medical School, and Clinical Center, University of Pécs, and the Teaching Hospital Markusovszky, Szombathely, Hungary. The current pilot study sample cohort was balanced with regards to sex and lung cancer tissue type. The patient data and the histological classification of the samples analyzed are detailed in Table 1. In the present work, we used optimized sample preparation steps and analysis methods. The most critical part of the work is the tissue surface digestion of the FFPE tissue samples, as it is difficult to control the size of the digested area. To address this issue, the digested area is circumvented with a razor blade to control the surface area.

In the preparation step, the samples were fixed in 10% buffered formaldehyde and embedded in paraffin. Each piece of tissue was cut into three micrometer-thick sections and stained with hematoxylin-eosin for diagnostic evaluation. Further sections were cut into ten-micrometer-thick pieces that were not stained. For enzymatic digestion, dewaxing is required, which was performed by washing with the following solvents: xylene, ethanol-water mixtures, and 10 mM ammonium bicarbonate.

This work was approved by the Medical Research Council (TUKEB permit number: IV/2567-4/2020/EKU).

### 4.3. Chondroitin Sulfate Digestion on Tissue Surface

The digestion of chondroitin sulfate chains was performed using our previously developed methodology with the chondroitinase ABC enzyme [28] using the following aqueous solution: 20 mM Tris–HCl, 2.5 mM ammonium acetate, and 2 mU/μL chondroitinase ABC (pH = 7.6). The selectivity of chondroitinase ABC towards CS/DS only was provided by the used buffer. The enzyme solution was added in five cycles of 2-μL droplets onto the surface. During the digestion samples were incubated in a humidified box for 1 h at 37 °C in each cycle, and then a final 24 h of incubation was done. Extraction of the resulting disaccharides was performed with 2 μL 1% ammonium hydroxide solution via 5 cycles of repeated pipetting. The samples were dried down after digestion and stored at −20 °C until further use. The structure and description of Δ4,5-unsaturated CS disaccharides can be found in Supplementary Tables S3 and S4.

### 4.4. Heparan Sulfate Digestion on Tissue Surface

The digestion of heparan sulfate chains was performed based on a previously developed methodology [28]. The following digestion solution was used: 20 mM Tris–HCl, 2.5 mM Ca(OH)_2_, 10% glycerol, 0.5 mU/μL of heparin lyase I, 0.1 mU/μL of heparin lyase II, and 0.1 mU/μL of heparin lyase III. 2-μL droplets of the enzyme solution were added onto the surface in three cycles on the first day, then twice on the second day. In each cycle, the samples were incubated in a humidified box at 37 °C; then, an additional overnight was performed. Extraction of the resulting disaccharides was performed with 2 μL 1% ammonium hydroxide solution via 5 cycles of repeated pipetting.

### 4.5. Sample Cleanup with Combined Extraction Method (Cotton Wool and Graphite Solid-Phase Extraction)

A solid-phase extraction cleanup of the resulting CS and HS disaccharide mixtures was performed using a combined clean-up method. In order to reduce sample loss, a two-step SPE purification procedure has been introduced. During the first step, the highly polar components (highly sulfated disaccharides) are bound by cotton wool, while the unbound, less polar components that pass through are further purified by graphite SPE. The first step was carried out on cotton wool solid phase in centrifuge pipet tips. The samples were applied in 95% ACN (1% trifluoroacetic acid), the salts and contaminants were washed with 50 μL 95% ACN (1% trifluoroacetic acid), and then the disaccharides were eluted at 37 °C, 10 μL 1–5% NH_3_ solvent. The samples were then dried down and stored at −20 °C until further use. The effluent of the first step underwent a second step, where Pierce graphite resin was used in spin columns. The samples were applied in water, the salts and contaminants were washed with water, and then the disaccharides were eluted in 60:40 *v*/*v* H_2_O to acetonitrile (0.05% trifluoroacetic acid). The elution fractions of the first and second steps were united, then dried down and stored at −20 °C until further use.

### 4.6. Liquid Chromatography-Mass Spectrometry

The HPLC–MS analysis of HS samples was performed by a Waters Acquity I-class UPLC (Milford, MA) coupled to a Waters Select Series Cyclic Ion Mobility (Milford, MA, USA) mass spectrometer. The HPLC-MS measurement of CS was executed by a Waters nanoAcquity UPLC system (Milford, MA, USA) attached to a Waters Q-Tof Premier mass spectrometer (Milford, MA, USA). The chromatographic separation of CS and HS disaccharides was achieved on a self-packed GlycanPac AXH-1 capillary column (250 μm i.d.). For the separation of chondroitin sulfate disaccharides, the previously published method was used, while for heparan sulfate disaccharides, a slightly modified HPLC-MS method was used. The following eluents were used for the analysis: 10 mM ammonium formate in 75:25 *v*/*v* ACN:water (pH 4.4) (Solvent A) and 65 mM ammonium formate in 75:25 *v*/*v* ACN:water (pH 4.4) (Solvent B). The CS separation was carried out by the following 15-min salt gradient: start at 6% B and elevate Solvent B to 12% in 0.5 min, then to 60% in 4.5 min, then hold at 100% B for 4 min, and finally equilibrate using the initial composition for 5 min. For the analysis of HS, a 25-min gradient was used, starting from 0% B and holding for 0.1 min, then increasing solvent B to 100% in 0.1 min and holding for 10 min, then reducing solvent B to 0% in 0.5 min and holding for 14.5 min [29,30,32]. The instrument parameters were the following: low-flow ESI ion source, the capillary voltage was set to 1.9 kV, the cone voltage was 20 eV, and the temperature was 120 °C. In the disaccharide measurement, HS disaccharides were observed in MS1 mode, where the trap collision energy was 6 eV and the transfer was 3 eV. The CS was measured in MS1 and MS/MS modes, the mono-sulfated isomer pairs were fragmented with 32 eV to determine stereochemistry. HPLC-MS chromatograms of selected samples can be found in the Supplementary Materials (Figures S7 and S8). The measurement data were uploaded to the GlycoPOST database [39].

### 4.7. Data Evaluation and Interpretation

MS peaks were integrated with the QuanLynx add-in of a Waters MassLynx 4.2 software (Milford, MA, USA). Statistical analysis and data visualization were done using R 4.0.5 in RStudio 1.4.1106 [24]. The statistical evaluation process was ase follows: Normality and equality of variances were assessed using the Shapiro-Wilk test and the Levene test, respectively, for both multiple and two-group comparisons. For multiple group comparisons, ANOVA, Welch-ANOVA, or Kruskal-Wallis tests were used, while for two-group comparisons, we used Student’s *t*-tests, Welch *t*-tests, and Wilcoxon rank sum tests based on the outcomes of the normality and variance equality tests. The Kolgomorov-Smirnov test was used to compare the distribution of HS sample groups. False discovery rates were controlled for all two-group and multiple-group comparisons separately at 5% using the Benjamini-Hochberg method. The gplots package and base R were used to create plots. Principal component analysis was conducted using the prcomp function with variable scaling and default settings, and hierarchical clustering was performed using the heatmap.2 function with Ward’s clustering method “ward.D2” from the hclust function.

## 5. Conclusions

In the present pilot study, the heparan sulfate and chondroitin sulfate content of various lung tumor phenotypes and corresponding adjacent normal tissues were analyzed. HPLC-MS disaccharide analysis was performed following on-surface digestion and SPE purification. Several significant differences in glycosaminoglycan content and sulfation were observed between tumor and tumor adjacent samples. Comparing the lung tumor phenotypes, the changes were not as striking. The total quantity of CS was doubled in tumor samples, while the total content of HS did not change significantly. The average degree of sulfation was significantly increased in all tumor phenotypes investigated. The 6-*O*-/4-*O*-sulfation ratio increased in adenocarcinomas compared to other lung tumor phenotypes. Examining the sulfation of HS, the *O*-sulfated components showed an increase in tumor samples. Our results highlight the importance of investigating the role of GAGs in the development of lung cancer, as several alterations could be identified between tumor and tumor-adjacent tissue samples as well as between the lung tumor phenotypes. Further investigation of the detected changes in a larger cohort can lead to a more detailed understanding of the functions and significance of structural changes in GAG chains in tumor development. GAGs play an important role in several signaling processes and are of interest in other cancer types as well. However, when planning experiments, the interpatient variance should be taken into consideration when choosing a sufficiently large sample size. We propose future, larger-scale studies to identify significant cancer-related structural changes in heparan sulfate and chondroitin sulfate GAGs.

## Figures and Tables

**Table 1:** Summary of the examined samples and groups. CS: cohort for chondroitin sulfate measurements; HS: cohort for heparan sulfate measurements (the median age is shown in the table).

	No. of Patients	No. of Tumor Samples	No. of Tumor Adjacent Samples
Total No.	42	81 (CS:41, HS: 40)	72 (CS: 36, HS: 36)
Age	65.5 (54–79)	65.5 (54–79)	65 (54–75)
Male		44	40
CS	22	22	20
HS	22	22	20
Female		37	32
CS	19	19	16
HS	18	18	16
Histology			
Adenocarcinoma		22	18
CS	12	12	9
HS	10	10	9
Large cell carcinoma		20	18
CS	10	10	9
HS	10	10	9
Small cell lung carcinoma		20	20
CS	10	10	10
HS	10	10	10
Squamous cell carcinoma		19	16
CS	9	9	8
HS	10	10	8

## Data Availability

The data presented in this study have been deposited in the GlycoPOST database under the accession number of GPST000337.

## Supplementary Materials

The following supporting information can be downloaded at: https://www.mdpi.com/article/10.3390/ijms24087050/s1.
