# Supplementary material for: Compositional Analysis of Glycosaminoglycans in Different Lung Cancer Types—A Pilot Study
Source: Int J Mol Sci. 2023 Apr 11;24(8):7050. doi: 10.3390/ijms24087050 (PMC10138872; doi:10.3390/ijms24087050)
Supplement: Supplementary file 1 [file ijms-24-07050-s001.zip › ijms-2285152-supplementary.pdf]

International Journal of *Molecular Sciences*

Supplementary information

Table S1. Statistically significant changes identified for chondroitin sulfate disaccharides.

The compared sample groups	Aspect of comparison	Tissue type	Number of tumor samples	Number of tumor adjacent samples	Statistical test	p-value	The degree of deviation (fold change)
All tumor and all adjacent	Total quantity		41	36	Wilcoxon	$<10^{-4}$	2.212
	D0a0 quantity		41	36	Wilcoxon	$<10^{-6}$	0.753
	D0a4 quantity		41	32	Wilcoxon	$<10^{-6}$	4.057
	D0a6 quantity		41	32	Wilcoxon	$<10^{-5}$	2.331
	Average sulfation		41	36	Wilcoxon	$<10^{-6}$	2.443
	D0a6/D0a4-sulfation ratio		41	36	Student t	$<10^{-2}$	0.830
Tumor and respective adjacent	D0a0 quantity	AC	12	9	Wilcoxon	$<10^{-2}$	0.767
		SCLC	10	10	Student t	<0.05	0.799
		SqCC	9	8	Student t	$<10^{-2}$	0.669
		LCC	10	9	Student t	<0.05	0.753
	D0a4 quantity	AC	12	6	Welch t	<0.05	3.241
		SCLC	10	10	Welch t	<0.05	3.513
		SqCC	9	8	Welch t	<0.05	5.905
		LCC	10	8	Wilcoxon	<0.05	4.625
	D0a6 quantity	AC	12	6	Wilcoxon	<0.05	4.168
		SqCC	9	8	Student t	<0.05	2.820
		LCC	9	8	Student t	<0.05	3.166
	Total quantity	AC	12	9	Wilcoxon	<0.05	1.991
		SqCC	9	8	Wilcoxon	<0.05	3.068
		LCC	10	9	Wilcoxon	<0.05	2.294
	Average sulfation	AC	12	9	Wilcoxon	$<10^{-2}$	3.238
		SCLC	10	10	Student t	<0.05	1.809
		SqCC	9	8	Student t	$<10^{-2}$	2.673
		LCC	10	9	Student t	<0.05	2.779
	D0a6/D0a4-sulfation ratio	SCLC	10	10	Student t	$<10^{-2}$	0.673
		SqCC	9	8	Student t	<0.05	0.719
		LCC	10	9	Student t	<0.05	0.786
Investigated tumor types	AC D0a6/D0a4-sulfation ratio	AC	12	0			
		SCLC	10	0	Student t	$<10^{-2}$	1.443
		SqCC	9	0	Student t	$<10^{-2}$	1.503
		LCC	10	0	Student t	$<10^{-2}$	1.432

Table S2. Statistically significant changes identified for heparan sulfate disaccharides.

The compared sample groups	Aspect of comparison	Number of tumor samples	Number of tumor adjacent samples	Statistical test	p-value	The degree of deviation (fold change)
All tumor and all adjacent	D2A0+D0A6 quantity	37	31	Wilcoxon	$<10^{-2}$	1.886
	D2S0+D0S6 quantity	40	36	Wilcoxon	<0.05	0.796
	Mono *N*/*O*-sulfation	36	31	Wilcoxon	$<10^{-2}$	0.599
	Di *N*/*O*-sulfation	40	36	Wilcoxon	<0.05	0.952
	Total *N*/*O*-sulfation	40	36	Student t	$<10^{-2}$	0.759

Table S3. The molecular structure, the traditional name, the Lawrence code and the analyzed m/z values of the HS disaccharides. The D2A0/D0A6 and D2S0/D0S6 isomers were not differentiated in this study.

Disaccharide structure	Traditional name	Lawrence code	m/z (−) mode
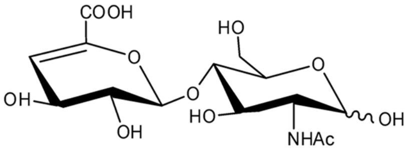	ΔHexA-GlcNAc	D0A0	378.10
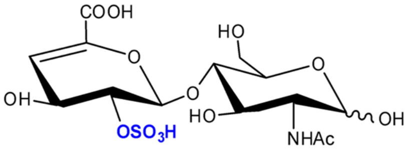	ΔHexA2S-GlcNAc	D2A0	458.10<br>247.50
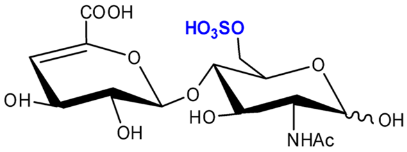	ΔHexA-GlcNAc6S	D0A6	458.10<br>247.50
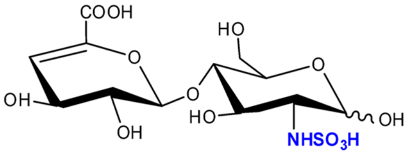	ΔHexA-GlcNS	D0S0	416.10<br>438.10<br>207.50
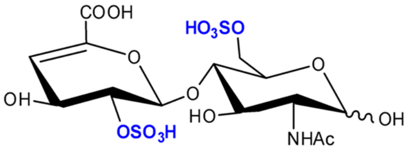	ΔHexA2S-GlcNAc6S	D2A6	538.05<br>560.05<br>576.00<br>286.50
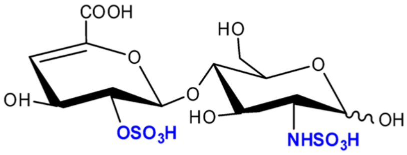	ΔHexA2S-GlcNS	D2S0	496.00<br>416.10<br>247.50

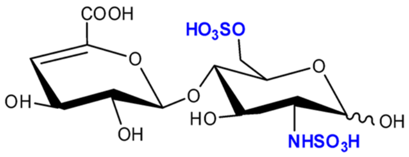	ΔHexA-GlcNS6S	D0S6	496.00<br>416.10<br>247.50
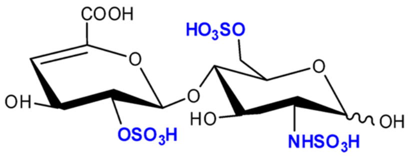	ΔHexA2S-GlcNS6S	D2S6	576.00<br>306.50<br>298.50<br>247.50<br>191.30

Table S4. The molecular structure, the traditional name, the Lawrence code and the analyzed m/z values of the CS disaccharides. The D0a4 and D0a6 disaccharides are positional isomers and their differentiation was made via MS/MS transition.

Disaccharide structure	Traditional name	Lawrence code	m/z (–) mode	MS/MS transition
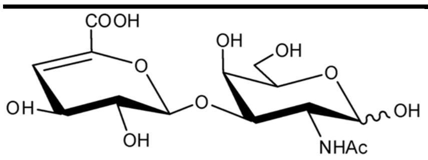	ΔHexA-GalNAc	D0a0	378.10	
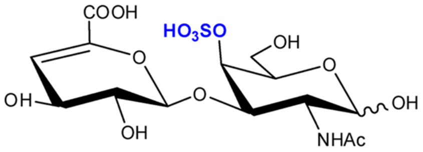	ΔHexA-GalNAc4S	D0a4	458.10<br>247.50	300.10
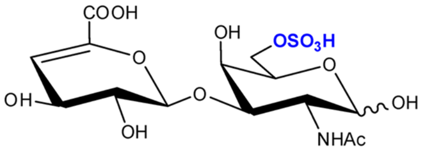	ΔHexA-GalNAc6S	D0a6	458.10<br>247.50	282.10
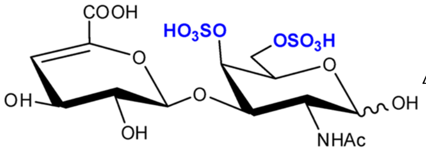	ΔHexA-GalNAc4S6S	D0a10	538.05<br>560.05<br>576.00<br>268.50	

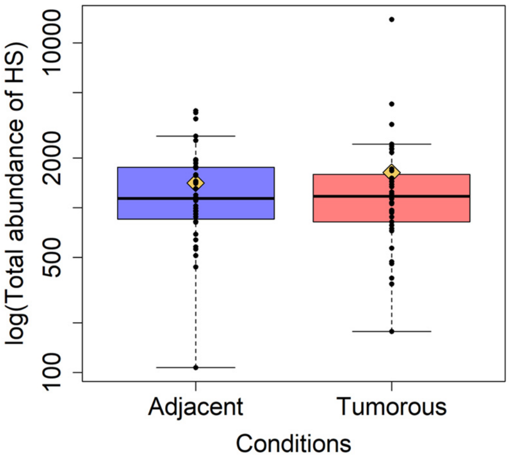

Figure S1. The total quantity of HS disaccharides in all the tumor and all the tumor adjacent samples. There is no significant change between the two sample groups.

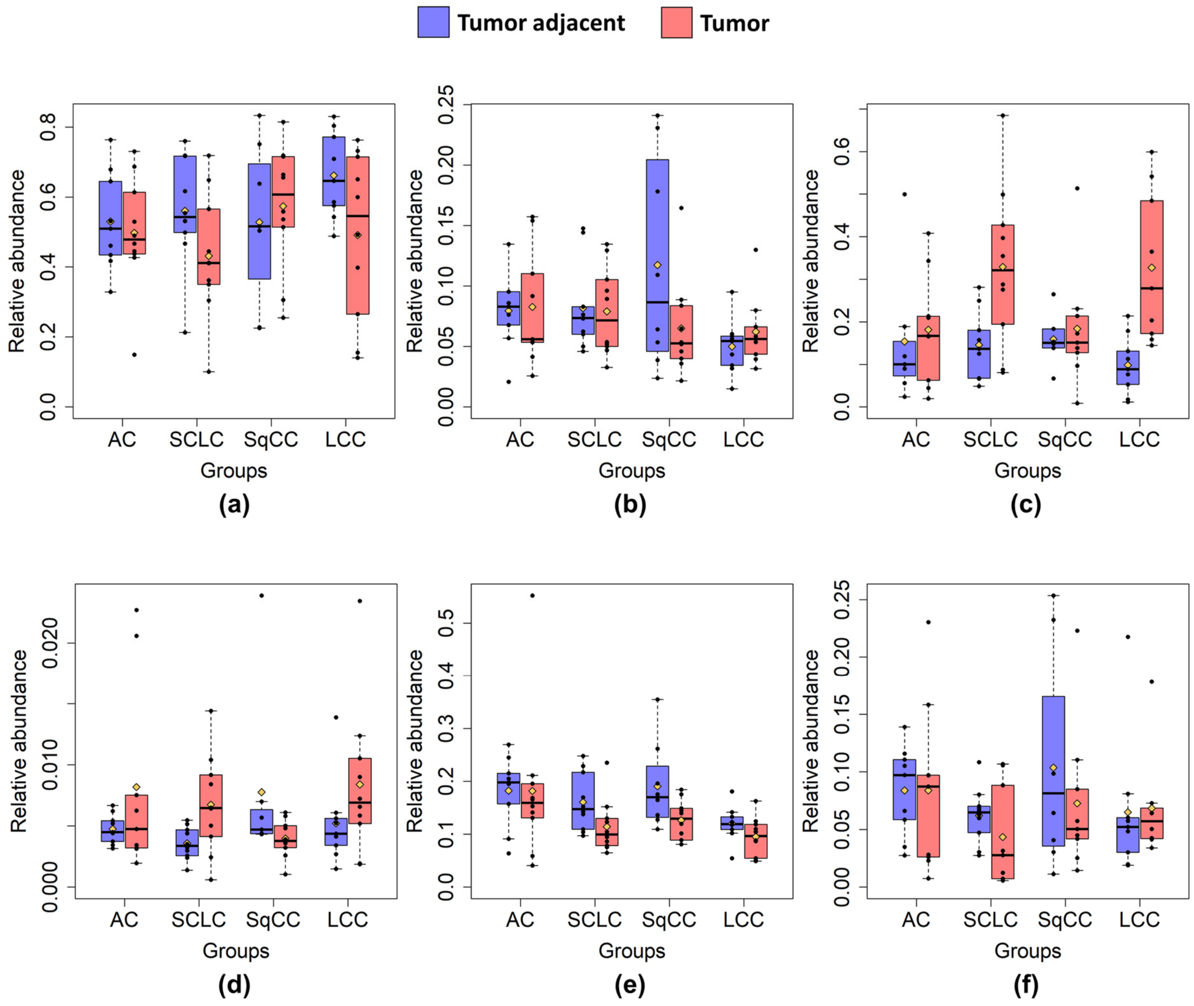

Figure S2. HS sulfation characteristics between tumor and tumor adjacent samples. (a): D0A0 relative abundance; (b): D0S0 relative abundance; (c): D02A0+D0A6 relative abundance; (d): D2A6 relative abundance; (e): D2S0+D0S6 relative abundance; (f): D2S6 relative abundance. Blue boxes represent tumor adjacent normal samples, while red boxes represent tumor samples. (AC: adenocarcinoma, SCLC: small cell lung cancer, SqCC: squamous cell carcinoma, LCC: large cell carcinoma).

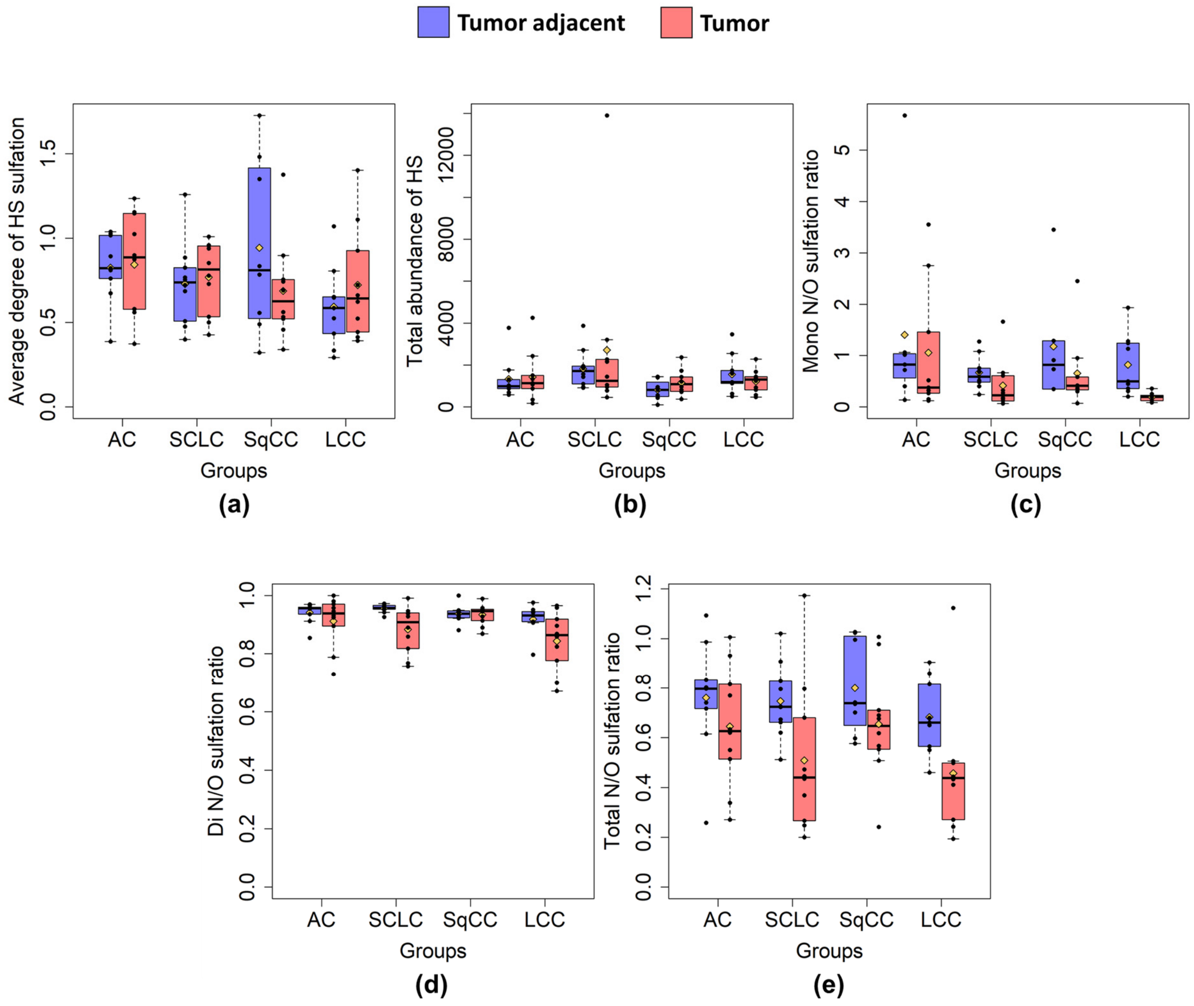

Figure S3. HS sulfation characteristics between tumor and tumor adjacent samples. (a): average sulfation of HS; (b): total quantity of HS; (c): mono-N/O-sulfation ratio; (d): di-N/O-sulfation ratio; (e): total N/O-sulfation ratio of HS. Blue boxes represent tumor adjacent normal samples, while red boxes represent tumor samples. (AC: adenocarcinoma, SCLC: small cell lung cancer, SqCC: squamous cell carcinoma, LCC: large cell carcinoma).

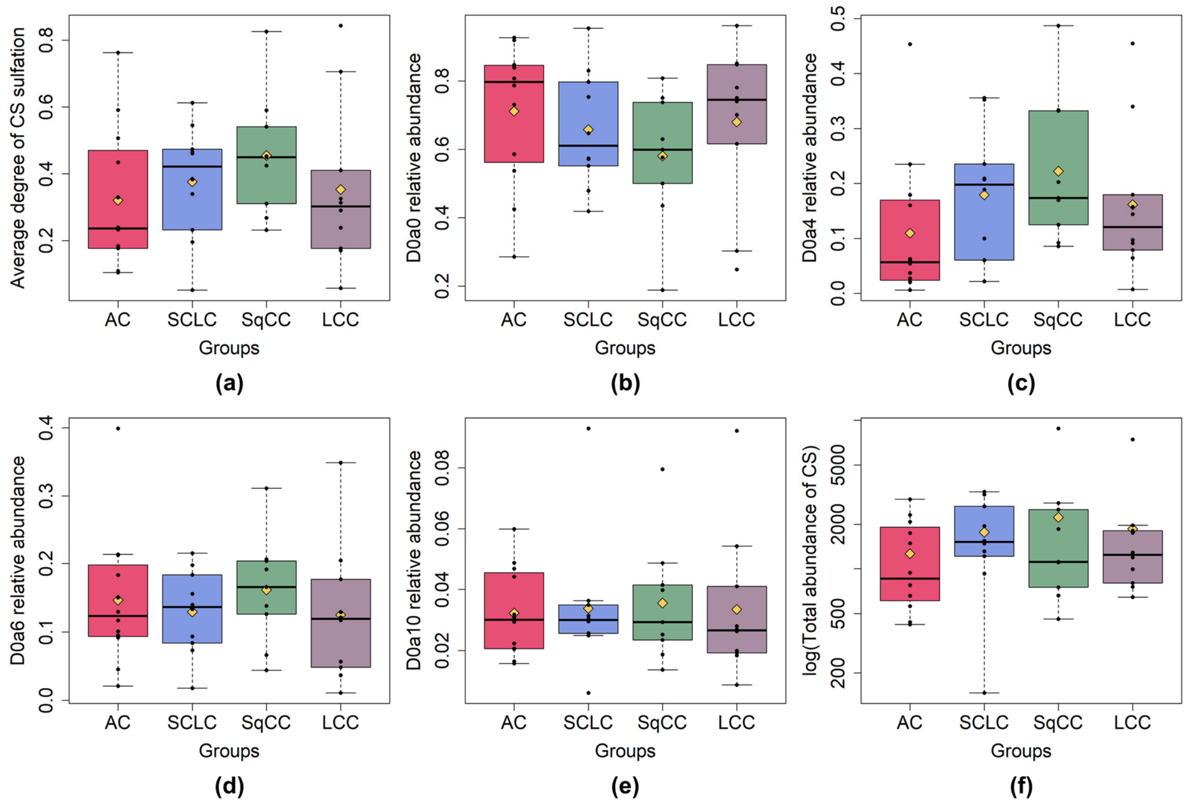

Figure S4. CS sulfation characteristics between tumor types. (a): average degree of sulfation; (b): D0a0 relative abundance, (c): D0a4 relative abundance, (d): D0a6 relative abundance, (e): D0a10 relative abundance, (f): total CS abundance. (AC: adenocarcinoma, SCLC: small cell lung cancer, SqCC: squamous cell carcinoma, LCC: large cell carcinoma).

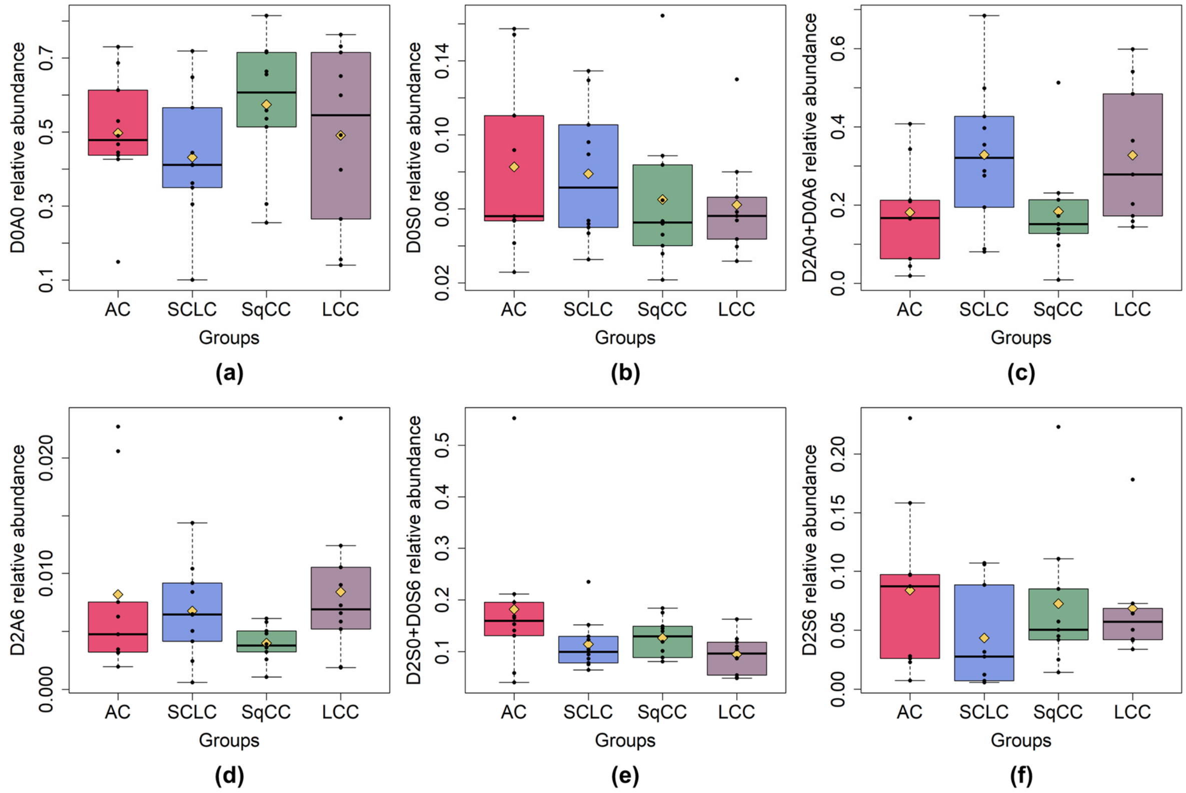

Figure S5. HS relative abundances between tumor types. (a): D0A0; (b): D0S0, (c): D0A0+D0A6, (d): D2A6, (e): D2S0+D0S6 (f): D2S6 relative abundance. (AC: adenocarcinoma, SCLC: small cell lung cancer, SqCC: squamous cell carcinoma, LCC: large cell carcinoma).

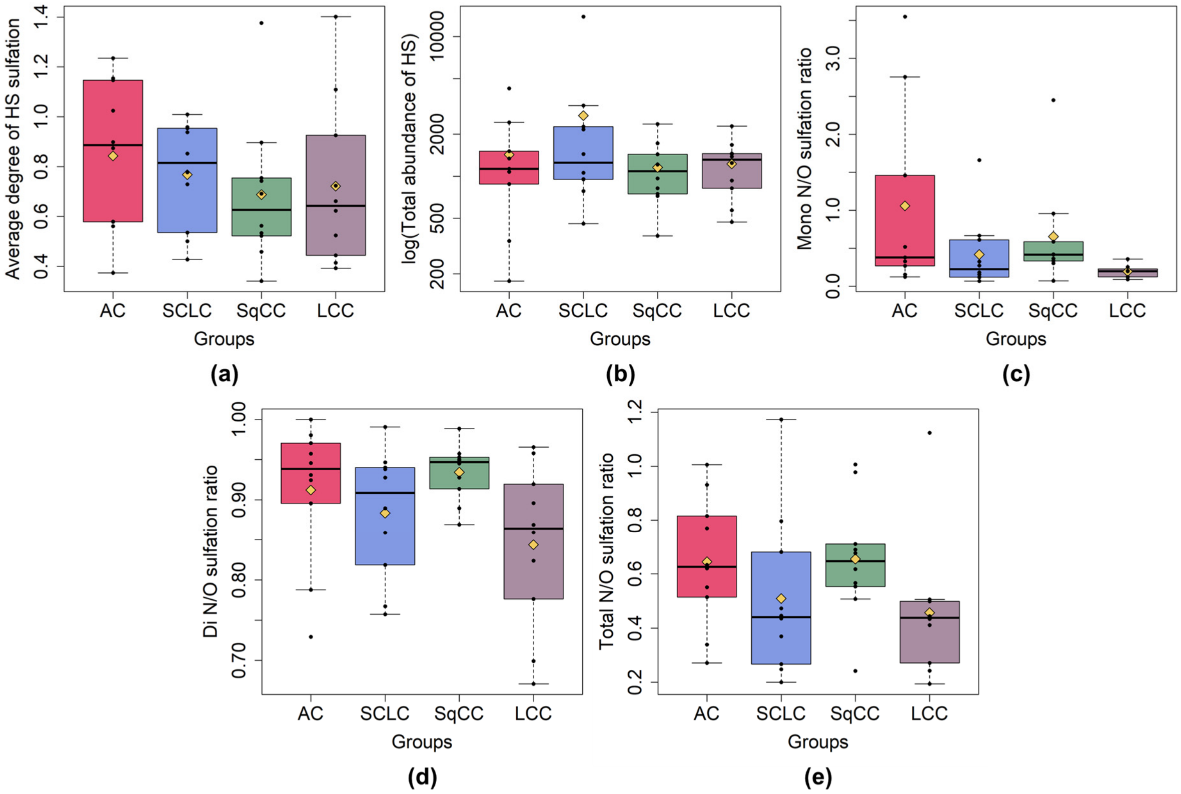

Figure S6. HS sulfation characteristics between tumor types. (a): average degree of sulfation; (b): total HS abundance, (c): mono-N/O- sulfation ratio, (d): di-N/O- sulfation ratio, (e): total-N/O-sulfation ratio. (AC: adenocarcinoma, SCLC: small cell lung cancer, SqCC: squamous cell carcinoma, LCC: large cell carcinoma).

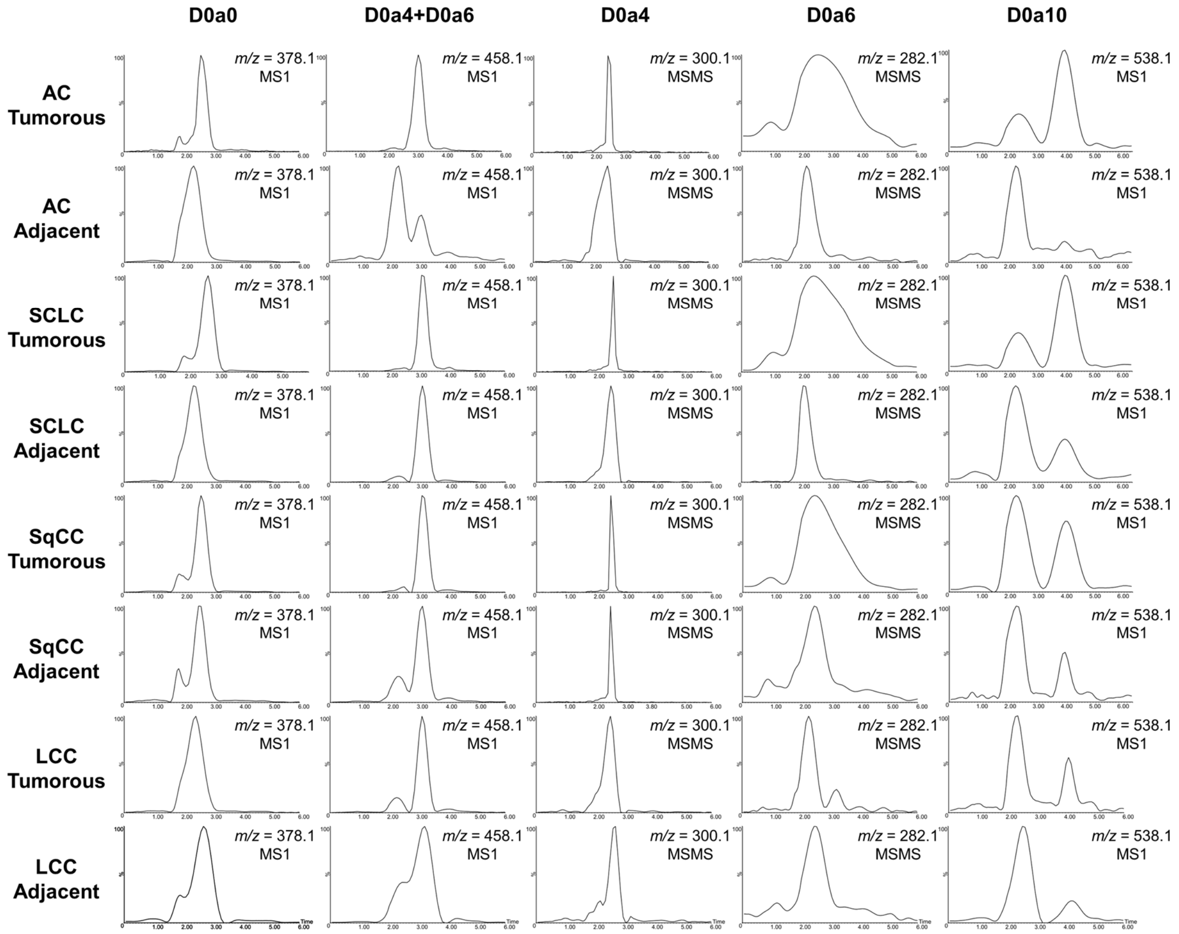

FigureS7. Representative extracted ion chromatograms of CS disaccharides of the investigated LC tumor types and tumor adjacent normal regions. (AC: adenocarcinoma, SCLC: small cell lung cancer, SqCC: squamous cell carcinoma, LCC: large cell carcinoma).

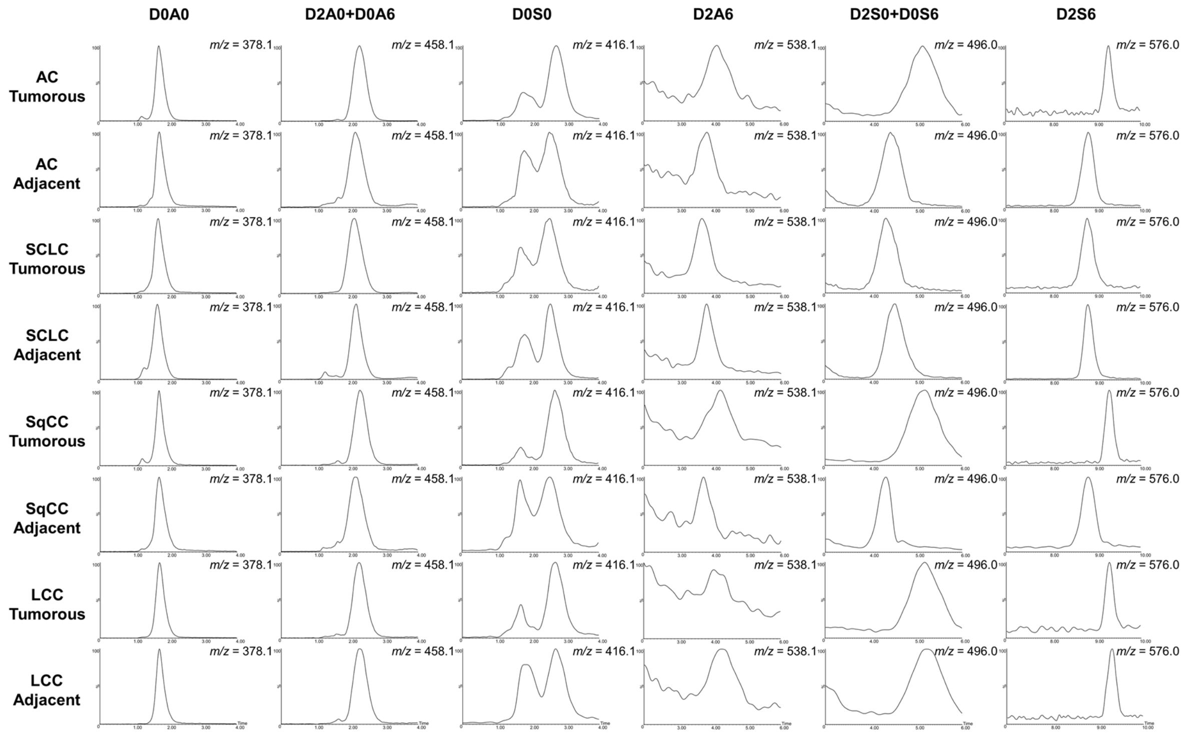

Figure S8. Representative extracted ion chromatograms of HS disaccharides of the investigated LC tumor types and tumor adjacent normal regions. (AC: adenocarcinoma, SCLC: small cell lung cancer, SqCC: squamous cell carcinoma, LCC: large cell carcinoma).
